# A GHRHR founder mutation causes isolated growth hormone deficiency type IV in a consanguineous Pakistani family

**DOI:** 10.3389/fendo.2023.1066182

**Published:** 2023-03-07

**Authors:** Safeer Ahmad, Muhammad Zeeshan Ali, Sumra Wajid Abbasi, Safdar Abbas, Iftikhar Ahmed, Shakil Abbas, Shoaib Nawaz, Mubarak Ziab, Ikhlak Ahmed, Khalid A. Fakhro, Muzammil Ahmad Khan, Ammira Al-Shabeeb Akil

**Affiliations:** ^1^ Gomal Centre of Biochemistry and Biotechnology, Gomal University, D.I. Khan, Khyber Pakhtunkhwa, Pakistan; ^2^ Department of Biological Sciences, National University of Medical Sciences, Rawalpindi, Punjab, Pakistan; ^3^ Laboratory of Genomic Medicine-Precision Medicine Program, Sidra Medicine, Doha, Qatar; ^4^ Department of Human Genetics, Precision Medicine of Diabetes Prevention Program, Sidra Medicine, Doha, Qatar; ^5^ Department of Genetic Medicine, Weill Cornell Medical College-Doha, Doha, Qatar; ^6^ College of Health and Life Sciences, Hamad Bin Khalifa University, Doha, Qatar

**Keywords:** isolated growth hormone deficiency (IGHD4), Pakistani family, whole-exome sequencing, GHRHR, modeling, docking and simulation

## Abstract

**Background:**

Isolated growth hormone deficiency (IGHD) is caused by a severe shortage or absence of growth hormone (GH), which results in aberrant growth and development. Patients with IGHD type IV (IGHD4) have a short stature, reduced serum GH levels, and delayed bone age.

**Objectives:**

To identify the causative mutation of IGHD in a consanguineous family comprising four affected patients with IGHD4 (MIM#618157) and explore its functional impact *in silico*.

**Methods:**

Clinical and radiological studies were performed to determine the phenotypic spectrum and hormonal profile of the disease, while whole-exome sequencing (WES) and Sanger sequencing were performed to identify the disease-causing mutation. *In-silico* studies involved protein structural modeling and docking, and molecular dynamic simulation analyses using computational tools. Finally, data from the Qatar Genome Program (QGP) were screened for the presence of the founder variant in the Qatari population.

**Results:**

All affected individuals presented with a short stature without gross skeletal anomalies and significantly reduced serum GH levels. Genetic mapping revealed a homozygous nonsense mutation [NM_000823:c.G214T:p.(Glu72*)] in the third exon of the growth-hormone-releasing hormone receptor gene *GHRHR* (MIM#139191) that was segregated in all patients. The substituted amber codon (UAG) seems to truncate the protein by deleting the C-terminus GPCR domain, thus markedly disturbing the GHRHR receptor and its interaction with the growth hormone-releasing hormone.

**Conclusion:**

These data support that a p.Glu72* founder mutation in *GHRHR* perturbs growth hormone signaling and causes IGHD type IV. *In-silico* and biochemical analyses support the pathogenic effect of this nonsense mutation, while our comprehensive phenotype and hormonal profiling has established the genotype–phenotype correlation. Based on the current study, early detection of *GHRHR* may help in better therapeutic intervention.

## Introduction

Isolated growth hormone deficiency (IGHD) is a condition characterized by growth retardation and development failure in affected children as a result of reduced growth hormone (GH) levels. It is estimated that between 1:3,480 and 1:10,000 live births are affected by IGHD (1–4). There are four IGHD types, IGHD I–IV, differentiated by their clinical spectrum, inheritance pattern, and associated genetic factors. Two IGHD I subtypes, IGHD IA (MIM# 262400) and IGHD 1B (MIM# 612781), are caused by *GH1* (MIM# 139250) and *GHRHR* (MIM# 139191) gene defects, respectively. In addition, a mutation at 17q23.3 on *GH1* underlies both IGHD 1B (MIM# 617281) and IGHD II (MIM# 173100). IGHD III (MIM# 307200) is caused by a genetic defect in *BTK* (MIM# 300300) located on Xq22, and IGHD IV (MIM# 618157) is caused by a genetic defect in *GHRHR* (MIM# 139191) located on 7p14.3. IGHD IA and IV occur most frequently, while types II and III are rare (OMIM database, accessed on 20 August 2022).

Both IGHD types IA and IB are autosomal recessive conditions characterized by short stature. In type IA, there is an absence of serum GH, and those affected produce anti-GH antibodies after GH treatment (5, 6). In type IB, there are low (but detectable) serum GH levels and no evidence of antibody production after GH treatment (7). IGHD type II, however, is an autosomal dominant condition; as with type IB, low serum GH levels are detectable and no anti-GH antibodies are produced upon GH treatment (8). IGHD type III usually segregates in an X-linked manner and is often associated with agammaglobulinemia (9). IGHD type IV is a recessive condition characterized by early and severe growth failure. Those affected exhibit a reduced GH response to various provocation tests (e.g., tests for determining growth hormone level) and low insulin-like growth factor-I (IGF1) and IGF-binding protein-3 (IGFBP3) levels, but a good response to GH treatment. At the cellular level, we know that human GH binds to human growth receptor (GHR) molecules and induces signal transduction through receptor dimerization (10). When GHRH interacts with its corresponding transmembrane domains on somatotropic (GH-producing) cells, a G protein-mediated interaction with ion channels causes an increase in intracellular cAMP accumulation, which ultimately promotes GH release from secretory granules (10–12). Indeed, elevated cAMP causes protein kinase A to phosphorylate and activate CREB (13, 14), whose target genes include the pituitary-specific transcription factor *Pit-1* (also known as *GHF-1*) (15–17). Pit-1 is a prototypic POU domain protein that is required for the proper regulation of *GH* gene activity in somatotropic cells, thereby providing a pathway by which a GHRH signal can lead to increased pituitary GH synthesis. Somatostatin, an inhibitory peptide, is thought to interact with this same signaling pathway *via* G protein-mediated suppression of the cAMP pathway (18, 19). Indeed, the malfunctioning or underexpression of endogenous CREB protein in pituitary somatotropic cells causes somatotroph hypoplasia and dwarfism in mice (20). It is now clear that any disruption to this multistep GH signaling pathway can result in GH deficiency and ultimately lead to short stature and various other clinical problems.

Here, we analyzed a Pakistani family comprising four affected individuals with IGHD4. Whole-exome sequencing in this family revealed a nonsense mutation NM_000823:c.G214T:p.Glu72* in the third exon of *GHRHR*. The identified mutation presumably creates a premature terminator codon in the extracellular domain of the GHRHR and results in the synthesis of a truncated and non-functional receptor. *In-silico* findings and biochemical analysis support the pathogenic effect of the reported nonsense mutation.

## Methods

### Study design, declarations, and approvals

This study was approved by the Ethical Review Board of Gomal University, Dera Ismail Khan, Pakistan. Informed consent to perform genetic, molecular, and clinical analyses and to publish patient data and images was obtained from all study participants. The family was identified in Tehsil Paroa of District Dera Ismail Khan, Pakistan, through a local street-to-street survey. The genealogy was ascertained to assess the mode of disease inheritance and determine the level of consanguinity between the parents. Then, the blood samples were collected, and DNA was isolated using a GeneJET Genomic DNA purification kit (Thermo Fisher Scientific, USA, Cat# K0721), according to the manufacturer’s instructions.

### Whole-exome sequencing and data analysis

All the patients (V-4, V-10, V-11, V-12) were siblings and exhibited the same phenotype (**Table 1**). Therefore, due to the high probability of harboring common genetic variants, WES was performed on two randomly selected patients (V-4 and V-10). A sequencing library was constructed using an xGen Exome Research Panel v2.0 Kit (Integrated DNA Technologies, Coralville, IA, USA) and sequenced on a NovaSeq 6000 (Illumina, San Diego, CA, USA). The base call files were converted to FASTQ files using bcl2fastq v2.20.0.422 (https://support.illumina.com/sequencing/sequencing_software/bcl2fastq-conversion-software.html). The sequence reads in the FASTQ files were aligned to the human reference genome (GRCh37/hg19) using BWA-mem 0.7.17 (arXiv:1303.3997v2 [q-bio.GN]) to generate BAM files. The BAM files were further processed for variant calling to generate VCF files using GATK v.3.8 (21). Finally, variant annotation and interpretation was performed using the EVIDENCE tool developed by “3billion” (South Korea) (22). To evaluate the authenticity of these predictions, variant annotation was carried out in parallel using wANNOVAR (https://wannovar.wglab.org/index.php) (23) and VariantStudio software (version 3.0) with the reference assembly “hg19.” Variant prioritization was phenotype driven (i.e., based on the disease diagnosis), and previously reported genes were used as the training set (retrieved from the OMIM database, accessed on 20 August 2022). Primarily, the genes reported for IGHD were screened for the presence of possible candidate variants before the variant analysis was extended exome-wide to identify protein-disrupting mutations. For candidate variant prioritization, the analysis focused on filtering homozygous, missense and indel variants, and low-frequency and protein-encoding alleles. Finally, splice variants and untranslated region (UTR) variants were also investigated to exclude their possible involvement.

**Table 1:** Anthropometric, hormone level, and general phenotypic measurements in affected individuals with familial IGHD-associated dwarfism.

Patient ID	V-4	V-10	V-11	V-12
Sex	Female	Male	Male	Male
Age (years)	37	26	11	12
Height (cm)	114	119	121	127
Weight (kg)	20	26	24	26
Stature (with reference to the average normal population)^a^	−7.6 SD (short stature)	−8.0 SD (short stature)	−7.7 SD (short stature)	−6.9 SD (short stature)
Growth hormone level (ref: 2.0–5.0 ng/ml)	<0.05	<0.05	0.08	0.1
Digit development	Normal	Normal	Normal	Normal
Dentition	Delayed	Delayed	Delayed	Delayed
Puberty onset	Delayed	Delayed	Delayed	Delayed
Voice pitch^b^	High	High	High	High
Frontal bossing	No	No	No	No
Obesity	No	No	No	No
General physique	Normal	Normal	Normal	Normal
Polydactyly	No	No	No	No
Clinodactyly	No	No	No	Yes
Brachydactyly	No	No	No	No
Split hand-foot malformation	No	No	No	No
Abnormality of the axial skeleton	No	No	No	No
Abnormality of the appendicular skeleton	No	No	No	No
Structure of the jaw	Normal	Normal	Normal	Normal
Neurological findings	Normal	Normal	Normal	Normal

Ref, reference range.

a SimulConsult was used to determine the population-compared stature (URL: https://simulconsult.com/resources/).

b The assessment was based on the general observation of voice, i.e., it could either be a child-like or adult-like voice.

### Preliminary computational screening

Before segregation analysis and 3D protein modeling, primary pathogenicity validation parameters were assessed. Initially, the biological significance of candidate variants was predicted using VarSome (24), which integrates multiple online pathogenicity prediction tools in a single platform. The gnomAD database (https://gnomad.broadinstitute.org/) was then consulted to assess the population allele frequency. To validate the clinical significance of identified candidate variants [as per ACMG guidelines (https://www.acmg.net/)], InterVar (25), VarSome (https://varsome.com/), and ClinVar databases (https://www.ncbi.nlm.nih.gov/clinvar/) were explored. Being a receptor protein, the integrity of transmembrane domains was assessed and visualized using TMHMM (https://services.healthtech.dtu.dk/service.php?TMHMM-2.0), Protter (https://wlab.ethz.ch/protter/start/), and Phobius (https://phobius.sbc.su.se/) prediction tools.

### Segregation analysis

To validate the segregation of the candidate variant(s) with the disease phenotype, Sanger DNA sequencing was performed on DNA samples provided by all available family members: III-1, IV-1, V-4, V-6, and V-10. The Primer3web tool (version 4.1.0) (26) was used to design suitable primers, and the mutation analysis was performed using BioEdit software (version 7.1) (27, 28).

### Biomedical analysis

To assess receptor functionality and GH deficiency, a clinical test for serum GH level was performed for two affected participants (V-10 and V-11). Blood samples (5 ml each) were collected from both the affected individuals under fasting conditions and referred to the CAP-certified (College of American Pathologists) laboratory at Aga Khan University Hospital, Karachi, Pakistan. X-ray images of the hands and feet were taken for all affected individuals to identify any limb abnormalities, e.g., bone degeneration (rheumatoid arthritis) and abnormal bone fusion (syndactyly).

### *In-silico* functional assays

Computational 3D structural modeling, protein–protein interaction analyses, and molecular dynamic simulation analyses were performed to predict the pathogenic effects of the identified variant(s) on the structure and function of the protein.

#### Protein structure prediction

Structural modeling of normal and mutant GHRHR was performed using the I-TASSER tool (29) and SWISS-MODEL (30). After structure prediction, 3D models of the wild-type and mutant GHRHR were superimposed to detect gross structural anomalies.

#### Molecular docking

ClusPro (31) was used to investigate the GHRH–GHRHR interaction. Namely, protein–protein docking of wild-type and mutant GHRHR was performed with its close functional interactor, GHRH, which was identified through the STRING matching database (32). Molecular visualization was performed using Chimera 1.13.1 (33) and LigPlot+ (version 2.1) (34).

#### Molecular dynamics simulation

AMBER package (version 16) (35) was used for molecular dynamic (MD) simulations of GHRHR models (wild type and mutant) and their docked complexes (35). All models were solvated in a rectangular box (8.0 Å) in the presence of counter ions to neutralize the system. Prior to MD simulations, each system was separately minimized, heated, and equilibrated. While keeping the protein model fixed, the studied systems were minimized. After minimization, the systems were heated and equilibrated at 300 K for 100 ps with a constant volume. Once the systems were equilibrated, all restraints were removed under constant pressure before running further equilibration steps at 300 K. MD simulation trajectory files were saved at 0.5 ps intervals. The PTRAJ/CPPTRAJ module (36) was used to analyze the saved MD simulation trajectory files, and Xmgrace (37) was used for visualization. The root mean square deviation (RMSD) and root mean square fluctuation (RMSF) were analyzed with the CPPTRAJ module. The RMSD values of both the wild-type and mutant structures were calculated using the starting structure as a reference frame and the deviation of the coordinates of a given set of atoms in a time interval. The RMSF was calculated to measure the fluctuations of each residue from their mean positions.

### Qatar Genome Program data analysis

The Qatar Genome Program (QGP) is a population-focused study that aims to generate a whole genome sequence for all participants in the Qatar Biobank (QBB) (38, 39). Qatar is a multicultural society where South Asian expats are considered to be one of the founder populations (40). The present study was conducted using information from ~15,000 QGP genomes that have been comprehensively phenotyped based on biomedical information deposited in the QBB. The whole genomes and phenotype data were investigated to determine the frequency of rs121918117 in the local Qatari population as well as their associated clinical phenotypes (including hormonal profiles and liver and kidney functions).

## Results

### Clinical findings of patients with IGHD type IV

**Figure 1:**
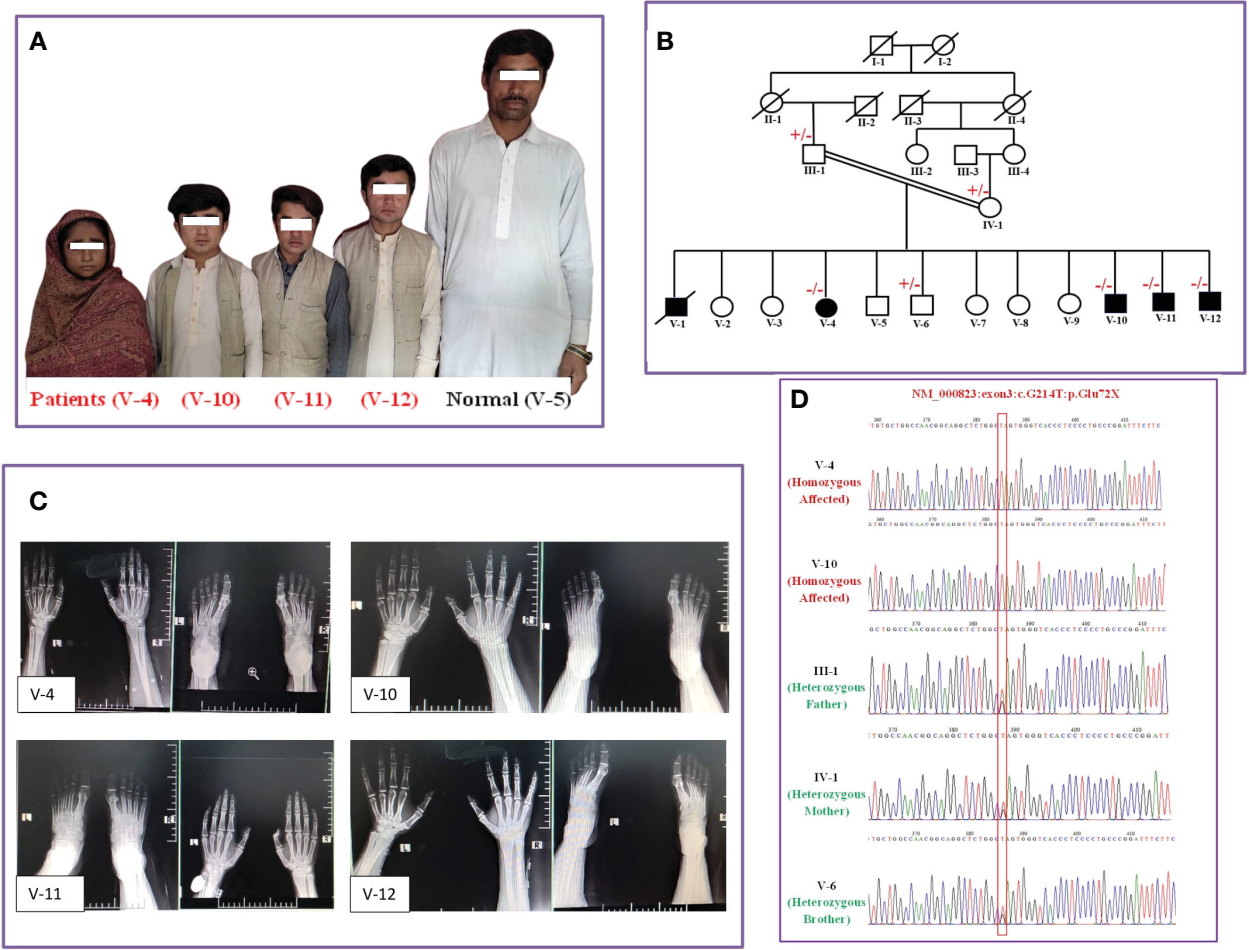
Genealogical analysis and clinical description of patients. **(A)** Photographs of normal (V-5) and affected individuals (V-4, V-10, V-11, V-12). **(B)** Genotype integrated family genealogy. **(C)** Radiographs of the hands and feet of affected patients. **(D)** Sequence chromatograms of affected patients (V-4 and V-10) and normal individuals, including the parents (III-1 and IV-1).

A five-generation consanguineous Pakistani family affected by IGHD type IV was recruited through a local survey. The family consisted of five affected individuals, namely, four surviving patients [one female (V-4) and three male patients (V-10, V-11, V-12) aged 37, 26, 11, and 12 years (**Figure 1A**), respectively] and one who died of an unrelated cause. The parents were first cousins with no previous history of IGHD and were asymptomatic with normal height, stature, and physique. The disease had emerged only in the most recent generation, and none of their predecessors exhibited this phenotype. All the patients had been born at full term with a normal delivery and no pregnancy complications. Genealogical analysis indicated a pattern of autosomal recessive disease inheritance (**Figure 1B**).

We first collected anthropometric, hormone, and general clinical measurements from affected individuals with familial IGHD-associated dwarfism (**Table 1**). We saw that all the surviving patients were affected by severe growth retardation and short stature (i.e., dwarfism) (**Figure 1A**). This finding supported the low or negligible serum GH levels, indicating abnormal GHRHR function. Specifically, all patients measured −6.9 to −8.0 SD below the mean height for the relevant age group in the healthy population (https://simulconsult.com/resources). By comparison, all unaffected individuals from general population had an average height of ~5.5 feet. Digit curvature, namely, bilateral 4/5 clinodactyly, was observed in the hands of V-12; no other digit anomalies such as polydactyly or brachydactyly were observed. Moreover, no other gross anomalies were detected by X-ray analysis of the appendicular or axial skeleton.

The body mass index (BMI) of all patients was in the normal range, and no one exhibited abdominal fat deposits. The head-to-body ratio was normal and non-indicative of micro- or macrocephaly; the thyroid gland examination was also normal. All patients exhibited a delay in the eruption of permanent dentition, yet the skull shape/size and jaw structure were normal in all patients, with no symptoms of hypoplastic maxilla. The radiological findings of the affected individuals revealed normally developed carpal/metacarpal and tarsal/metatarsal bones. There was no polydactyly, syndactyly, or brachydactyly of the hands or feet, thus suggesting normal digit development (**Figure 1C**).

The general clinical assessment of the patients did, however, indicate an underdevelopment of the body structure. All patients had a high-pitched voice. In medical examinations, no frontal bossing, depressed nasal bridge, or depressive behavior was observed. While fertility was not assessed in these patients, it was noted that all had reached puberty by 18 years of age (on average).

The findings of ophthalmologic, otorhinolaryngologic, neurologic, skeletal, and visceral organ examinations were normal. The patients had no current or previous history of diabetes, hypoglycemia, hypertension, cardiac problem, allergies (food or drug), dermal lesions, or autoimmune disorders.

### Molecular analysis found founder mutation in GHRHR

Having comprehensively assessed the clinical phenotypes of all four affected patients, we next performed a WES analysis to determine the genetic basis of the disease. Here, we identified a recurrent homozygous nonsense mutation [NM_000823:c.G214T:p.(Glu72*); (rs121918117)] in the third exon of the *GHRHR* gene. Sanger sequencing confirmed the co-segregation of the identified mutation with the disease phenotype in all patients (**Figure 1D**). This variant was not found in the unaffected family members or in our in-house database comprising an ethnically matched control population. The minor allele frequency of this allele in gnomAD was 0.0001755, with no known homozygote. According to a database survey, the variant was reported in South Asian regions only and not found in any European, American, Ashkenazi Jews, or East Asian communities, thus indicating that the variant is Asian-specific (allele count 34). Indeed, the (p.Glu72*) nonsense mutation has previously been mapped in three South Asian families (Indian, Sri Lankan, and Pakistani), which suggests a founder effect of this mutation in IGHD.

**Figure 2:**
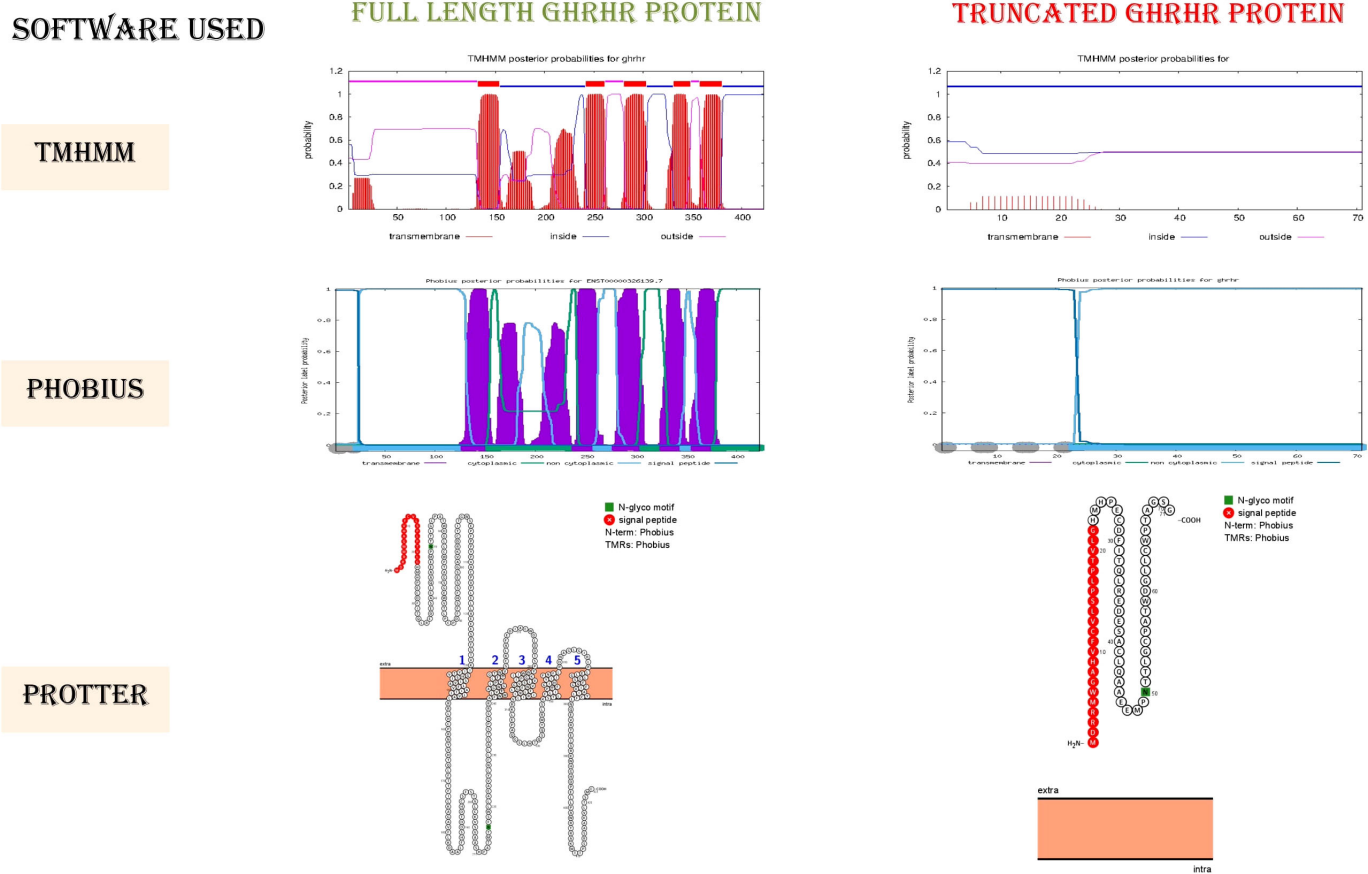
Membrane topology of GHRHR: images of the transmembrane topology of the GHRHR protein.

The ClinVar, VarSome, and InterVar databases reported rs121918117 as a pathogenic variant. Indeed, the premature stop codon presumably truncates the protein by deleting the C-terminus GPCR transmembrane (TM) domains and, in part, the GPCR-2 extracellular domains. According to TM domain integrity prediction, the truncated protein is unable to anchor within the membrane due to loss of all the downstream TM domains, causing loss of receptor function (**Figure 2**). We thus speculate that the premature stop codon and early truncation of the protein product caused by the identified nonsense mutation (p.Glu72*) might activate the nonsense-mediated mRNA decay pathway, resulting in low GH production in affected patients.

### Protein structural analysis

#### Molecular modeling of wild-type and mutant GHRHR proteins and hormone-receptor docking

**Figure 3:**
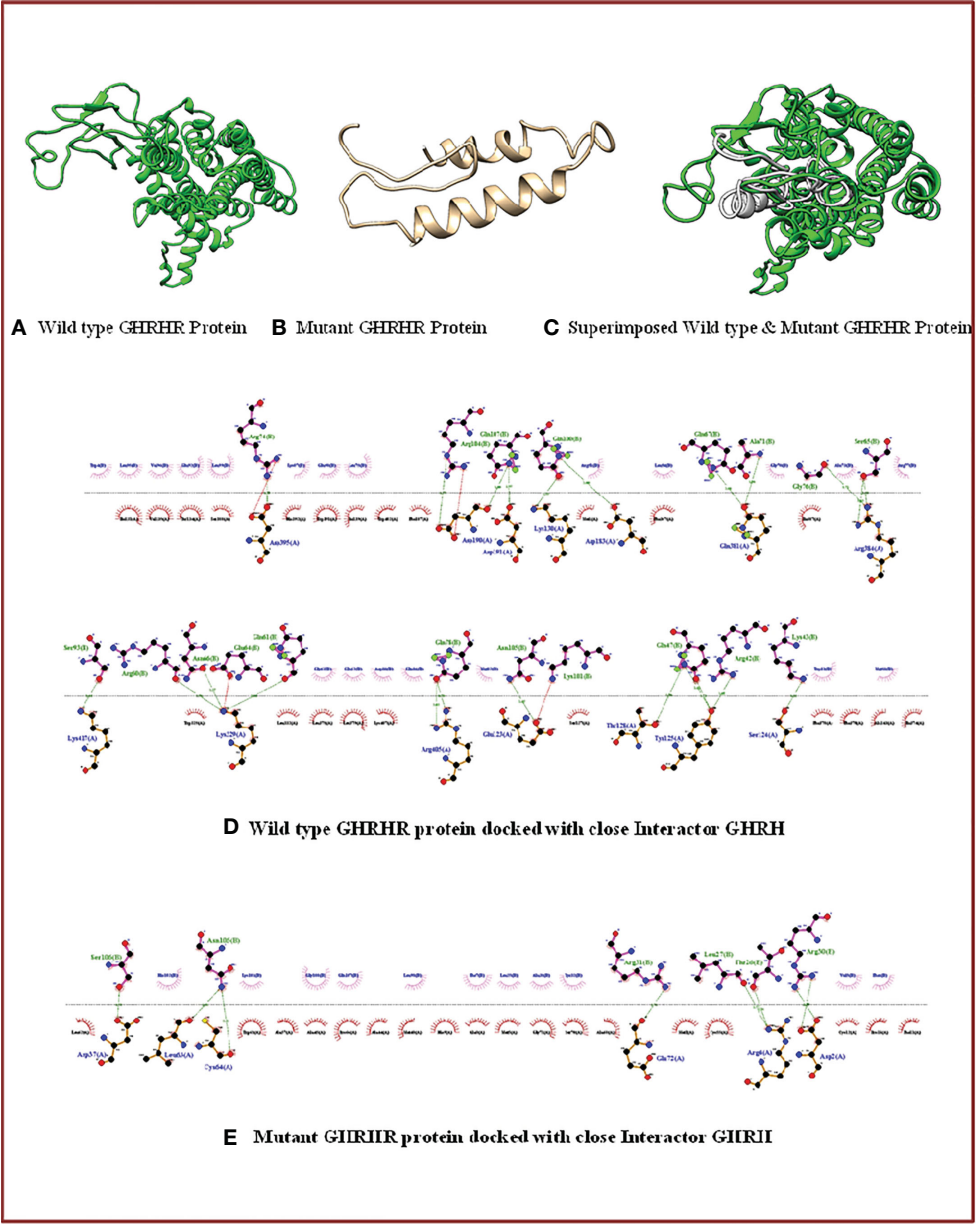
3D structure and docking properties of GHRHR: **(A–C)** Normal and mutant models of the GHRHR protein and their superimposed images. **(D, E)** Normal and mutant GHRHR proteins docked with a close interactor (GHRH).

We next aimed to determine the structure and interaction studies. We first found that superimposition of the 3D models of both wild-type and mutant GHRHR was not possible (**Figures 3A–C**), confirming that the c.G214T: p.Glu72* nonsense mutation causes structural distortion of GHRHR due to protein truncation.

We next monitored the interactions between the wild-type and mutant GHRHR receptor proteins with GHRH. Compared with the full-length wild-type protein, the truncated GHRHR protein showed a different interaction pattern with respect to residue numbers, residue identity, and bonding category (**Figures 3D, E**). The wild-type receptor could interact with GHRH *via* 14 amino acid residues (Asp395, Lys130, Asp183, Ser124, Arg384, Asp190, Asp191, Lys329, Arg405, Lys417, Glu123, Gln381, Thr128, and Tyr125) and 27 bonding forces involving 23 H-bonds and four salt bridges. By contrast, the mutant receptor docked with GHRH *via* only six amino acids (Leu63, Cys64, Asp37, Arg4, Asp2, and Glu72) and eight hydrogen bonds. These findings suggest that the mutation protein binds more strongly with the interactor compared to the wild-type GHRHR.

#### Findings on molecular dynamic simulation analysis

**Figure 4:**
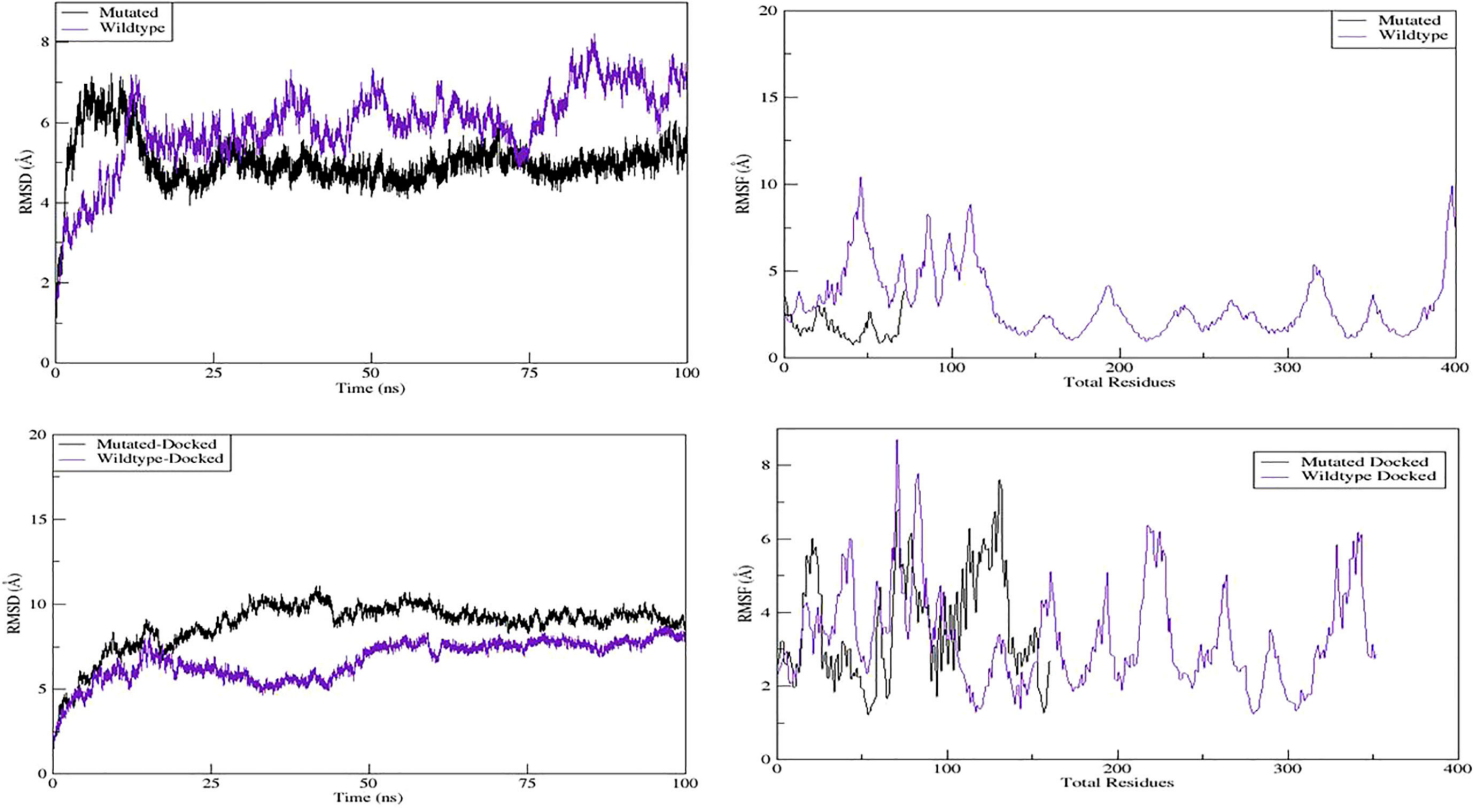
MD simulation properties: molecular dynamic simulation graphs of wild-type, mutant, and their docked complexes with GHRH.

Next, we evaluated the dynamic behavior of the wild-type and mutant GHRHR proteins and their interactions with GHRH by 100-ns MD simulation. We generated models of the wild-type–GHRHR–GHRH and mutant–GHRHR–GHRH complexes (**Figures 3D, E**, respectively). We determined the atomic positions of the modeled GHRHR protein structure and compared them with those of the mutant–GHRHR protein to examine the convergence and durability of the MD simulations. The trajectories of the MD simulations were analyzed in a multistep process to decode the backbone stability and residual flexibility. The RMSD was first calculated as a measure of the average distance between the backbone carbon alpha atoms in the overlay frames. The average distance between the backbone carbon alpha atoms of the overlay frames was then calculated, and the stability of the simulated C atoms of the protein was determined by plotting the modeled wild-type–GHRHR, mutant–GHRHR protein, wild-type–GHRHR–GHRH protein, and mutant–GHRHR–GHRH proteins as a function of time. We found evidence for a slightly greater deviation of the wild-type–GHRHR (RMSD mean 5.93364 Å, max. 8.2271 Å at 85,361 frames) than the mutant–GHRHR (RMSD mean 5.02231 Å, max. 7.2316 Å at 8,858 frames) (**Figure 4**, upper panel). In both the wild-type– and mutant–GHRHR, a constant displacement was observed in the first 25 ns of the MD simulation, indicating that the atoms were out of alignment and the structure was unstable. While the mutant–GHRHR showed minor fluctuations for the remaining simulation period, the wild-type–GHRHR–GHRH showed considerable deviations (RMSD mean 8.79908 Å, max. 8.79908 Å at 42,247 frames), indicating that the docked complex was stable after 60 ns (**Figure 4**, lower panel). By contrast, the RMSD plot for the mutant–GHRHR–GHRH revealed an unstable system that stabilized during the MD simulation and remained stable until the end. The plot showed considerable deviations (RMSD mean 6.73827 A, max. 8.7608 Å at 97,250 frames), indicating that the atoms were out of synchrony and the system was unstable. We also performed an RMSF analysis to assess the fluctuation of residues in the wild-type and mutant docked complexes. Here, the mutant structure showed much greater stability than the wild-type structure, which instead underwent marked fluctuations.

### Estimation of different binding free energies using MMGB/PBSA

We assessed the binding free energies of the wild-type and mutant GHRHR proteins complexed with GHRH. We calculated these based on the simulated trajectories using the MMGBSA and MMPBSA approaches. The net total energy for the wild-type–GHRHR–GHRH structure was −215.747, while that for the mutant–GHRHR–GHRH structure was −99.2622. In comparison to the MMGBSA approach, the MMPBSA net binding energy showed that the interaction energies of both systems were highly favorable and stable (**Table 2**).

**Table 2:** Calculation of binding free energies using MMGB/PBSA analysis.

	GB-normal	GB-mutant		PB-normal	PB-mutant
Complex					
Energy component	Average	Average	Energy component		Average
VDWAALS	−3,155.62	−1,061.13	VDWAALS	−3,155.62	−1,061.13
EEL	−28,758.908	−10,337.53	EEL	−28,758.91	−10,337.53
EGB	−6,074.956	−2,103.894	EPB	−5,976.96	−2,051.191
ESURF	203.5613	80.608	ENPOLAR	127.0985	52.8673
G gas	−31,914.528	−11,398.66	G gas	−31,914.53	−11,398.66
G solv	−5,871.3948	−2,023.286	G solv	−5,849.862	−1,998.324
TOTAL	−37,785.923	−13,421.94	TOTAL	−37,764.39	−13,396.98
Receptor					
VDWAALS	−2,325.1964	−330.9864	VDWAALS	−2,325.196	−330.9864
EEL	−21,591.286	−3,602.448	EEL	−21,591.29	−3,602.448
EGB	−5,949.3834	−1,480.209	EPB	−5,832.037	−1,438.954
ESURF	176.2588	38.5172	ENPOLAR	112.0205	25.7334
G gas	−23,916.482	−3,933.434	G gas	−23,916.48	−3,933.434
G solv	−5,773.1246	−1,441.692	G solv	−5,720.017	−1,413.22
TOTAL	−29,689.607	−5,375.127	TOTAL	−29,636.5	−5,346.655
Ligand					
VDWAALS	−555.6505	−603.0366	VDWAALS	−555.6505	−603.0366
EEL	−5,634.018	−5,790.622	EEL	−5,634.018	−5,790.622
EGB	−1,786.1916	−1,632.523	EPB	−1,767.252	−1,598.376
ESURF	65.4848	59.1637	ENPOLAR	44.7775	40.9695
G gas	−6,189.6685	−6,393.658	G gas	−6,189.669	−6,393.658
G solv	−1,720.7068	−1,573.359	G solv	−1,722.475	−1,557.407
TOTAL	−7,910.3753	−7,967.017	TOTAL	−7,912.143	−7,951.065
Differences (complex–receptor–ligand)					
VDWAALS	−274.7731	−127.1068	VDWAALS	−274.7731	−127.1068
EEL	−1,533.6038	−944.4587	EEL	−1,533.604	−944.4587
EGB	1,660.619	1,008.838	EPB	1,622.329	986.1388
ESURF	−38.1823	−17.0729	ENPOLAR	−29.6994	−13.8355
DELTA G gas	−1,808.3769	−1,071.566	DELTA G gas	−1,808.377	−1,071.566
DELTA G solv	1,622.4366	991.7649	DELTA G solv	1,592.63	972.3033
DELTA TOTAL	−185.9402	−79.8006	DELTA TOTAL	−215.747	−99.2622

### QGP data outcomes

To determine the frequency of rs121918117 (GHRHR) in the population, we leveraged the QGP database and identified two heterozygous individuals (QGP1 and QGP2) with an alternate allele frequency of 0.00006817. Both QGP1 and QGP2 were clinically normal and asymptomatic although their seated height was slightly reduced compared to that of the average value of the QGP-cataloged individuals. In terms of standing height, QGP1 was in the normal range, while QGP2 had a short stature (−0.5 SD). All other available health indicators were in the good and excellent categories, with the exception that QGP2 was classified as obese and QGP1 had systolic/diastolic BP and pulse rates tending to 89/62 mmHg and 68 beats per minute.

## Discussion

IGHD is an inherited condition of inadequate secretion of GH by the pituitary gland (41) that results in severe short stature (9). To date, 82 mutations in *GHRHR* have been identified in IGHD patients of multiple ethnicities. Of these mutations, six are nonsense, 12 are splice disrupting, 44 are missense, 16 are small indels, and four are regulatory mutations (42–46) (HGMD^®^ Professional 2022.2, assessed on August 2022) (**Table 3**). Here, we have added to the portfolio of GHRHR mutations underlying IGHD after performing a WES analysis of a large, consanguineous Pakistani family comprising four individuals suffering from IGHD4.

**Table 3:** List of reported mutations in the *GHRHR* gene.

Missense/nonsense mutations					
S. no.	cDNA position	Amino acid position	Exon number	Phenotype	Reference
1	c.11G*>*A	p.R4Q	1	Growth hormone deficiency	(47)
2	c.10C>T	p.R4W	1	Isolated growth hormone deficiency	(48)
3	c.29T>G	p.V10G	1	Growth hormone deficiency	(49)
4	c.47C>T	p.P16L	1	Isolated growth hormone deficiency	(48)
5	c.97C>T	p.Q33X	2	Growth hormone deficiency	(50)
6	c.128C>T	p.Q43X	2	Growth hormone deficiency	(45)
7	c.164G>T	p.C55F	3	Isolated growth hormone deficiency	(51)
8	c.244G>A	p.A57T	3	Increased tumor sensitivity/isolated growth hormone deficiency	(52)
9	c.190T>G	p.C64G	3	Growth hormone deficiency	(53)
10	c.211G>T	p.G71C	3	Isolated growth hormone deficiency	(54)
11	265G>T 214G>T	p.E72X	3	Growth hormone deficiency	(55) (56) (57)
12	c.236C*>*T	p.P79L	3	Growth hormone deficiency	(47)
13	c.281G>T	p.R94L	4	Growth hormone deficiency	(57)
14	c.280C>T	p.R94W	4	Growth hormone deficiency	(58)
15	c.281G>A	p.R94Q	4	Isolated growth hormone deficiency	(48)
16	c.335G>A	p.C112Y	4	Isolated growth hormone deficiency	(59)
17	c.428T>C	p.V121D	4	Isolated growth hormone deficiency	(60)
18	c.407G>T	p.G136V	5	Isolated growth hormone deficiency	(43)
19	c.410A>T	p.H137L	5	Growth hormone deficiency	(61)
20	c.418T>C	p.S140P	5	Isolated growth hormone deficiency	(51)
21	c.431T>A	p.L144H	5	Growth hormone deficiency	(61)
22	c.458C>A	p.A153D	5	Pituitary	(62)
23	c.481C>T	p.R161W	6	Growth hormone deficiency	(57)
24	c.537C>T	p.Y163Y	6	Isolated growth hormone deficiency	(60)
25	c.491T>C	p.V164A	6	Isolated growth hormone deficiency	(48)
26	c.495C>A	p.H165Q	6	Isolated growth hormone deficiency	(51)
27	c.507C>G	p.F169L	6	Isolated growth hormone deficiency	(48)
28	c.527C>T	p.A176V	6	Growth hormone deficiency	(63)
29	c.604T>C	p.C202R	7	Short stature	(64)
30	c.655C>A	p.A222E	7	Growth hormone deficiency	(61) (57)
31	c.728G>A	p.W243X	7	Isolated growth hormone deficiency	(48)
32	c.731G>A	p.W244X	7	Growth hormone deficiency	(65)
33	c.789C>T	p.L247L	7	Isolated growth hormone deficiency	(60)
34	c.758C>T	p.P253L	8	Isolated growth hormone deficiency	(59)
35	c.817A>G	p.T257A	8	Isolated growth hormone deficiency	(60)
36	c.776C>A	p.T259K	8	Isolated growth hormone deficiency	(48)
37	c.783G>A	p.V261V	8	Isolated growth hormone deficiency	(66)
38	c.838A>G	p.K264E	8	Isolated growth hormone deficiency	(60)
39	c.818G>C	p.W273S	9	Growth hormone deficiency	(57)
40	c.847T>C	p.W283R	9	Isolated growth hormone deficiency	(48)
41	c.998G>C	p.S317T	10	Isolated growth hormone deficiency	(60)
42	c.386A>G	p.K329E	11	Growth hormone deficiency	(45)
43	c.1037C>T	p.S330L	11	Isolated growth hormone deficiency	(60)
44	c.1071C>T	p.R357C	11	Growth hormone deficiency	(67)
45	c.1087G>C	p.G363R	12	Short stature	(64)
46	c.1102C>T	p.Q368X	11	Isolated growth hormone deficiency	(59)
47	c.1106G>T	p.G369V	12	Isolated growth hormone deficiency	(60)
48	c.1146G>A	p.E382E	12	Short stature	(46)
49	c.1160T>C	p.I387T	13	Isolated growth hormone deficiency	(48)
50	c.1265T>C	p.M422T	13	Major depressive disorder	(68)
Splice site mutations					
S. no.	Position	Intron number	Type	Phenotype	Reference
51	IVSI+1G>A	1	Splice donor	Growth hormone deficiency	(69)
52	IVS1+2T>G	1	Splice donor	Growth hormone deficiency	(70)
53	IVS2+3A>G	2	Splice donor	Isolated growth hormone deficiency	(43)
54	IVS3+1G>A	3	Splice donor	Growth hormone deficiency 1B	(45)
55	IVS7+1G>C	7	Splice donor	Growth hormone deficiency	(71)
56	IVS7−1G>A	7	Splice acceptor	Growth hormone deficiency	(72)
57	IVS8−30G>A	8	Splice acceptor	Isolated growth hormone deficiency	(66)
58	IVS8+1G>A	8	Splice donor	Pituitary dwarfism	(73)
59	IVS12−1G>A	12	Splice acceptor	Growth hormone deficiency 1B	(46)
60	IVS12+2T>A	12	Splice donor	Growth hormone deficiency	(74)
61	IVS3161−1G>A	3	Splice acceptor	Isolated growth hormone deficiency	(48)
62	IVS2+27G>T	2	Splice donor	Isolated growth hormone deficiency	(66)
Insertion/deletion mutations					
S. no.	cDNA position	Amino acid	Exon number	Phenotype	Reference
63	c.340delG	p.V114Cfs*16	4	Isolated growth hormone deficiency	(44)
64	c. 1140-1144del	p.N380Kfs*5	13	Isolated growth hormone deficiency	(61)
65	c.1120_1123delATCC	p.I374Sfs*9	11	Growth hormone deficiency	(58)
66	c.4bp del	p.373	12	Isolated growth hormone deficiency	(75)
67	c.380insC	p.C112Lfs*9	4	Isolated growth hormone deficiency	(60)
68	c.920insC	V308Gfs*79	10	Isolated growth hormone deficiency	(66)
69	g.30999250_ 31006943delinsAGAGATCCA	–	5" UTR/exon 1	Isolated growth hormone deficiency	(76)
70	c.271dupG	p.A91Gfs*13	4	Isolated growth hormone deficiency	(48)
71	c.674_677delinsGC TGTTGGCAGAAG	p.V225 Gfs*165	7	Isolated growth hormone deficiency	(48)
72	c.465−91_1105−119del5291	p.R156 Afs*15	5	Isolated growth hormone deficiency	(48)
73	c.(?_−48)_(57 + 1_58−1)del	–	2	Isolated growth hormone deficiency	(48)
74	c.22_23insA	p.A8Dfs*22	1	Isolated growth hormone deficiency	(48)
75	c.1089_1093del	p.L364Ffs*21	11	Isolated growth hormone deficiency	(48)
76	c.597+153_883−273del	p.L201_V295del	7	Isolated growth hormone deficiency	(48)
77	c.-3166_58−2057del	–	Intron1	Isolated growth hormone deficiency	(77)
78	c.820_821ins	p.N274Afs*113	9	Congenital Hypopituitarism	(78)
Regulatory mutations					
S. no.	Position	Phenotype	Reference		
79	c.-261C>T	Breast cancer	(69)		
80	c.166T>C	Reduced promoter activity/short stature	(46)		
81	c.164T>C	Reduced promoter activity/short stature	(46)		
82	c.-124A>C	Growth hormone deficiency 1B	(45)		

Our WES and Sanger sequencing analysis revealed a nonsense *GHRHR* mutation [NM_000823:exon3:c.G214T:p.Glu72*] that segregated with the disease phenotype. This nonsense mutation results in the generation of a truncated protein devoid of the C-terminus membrane spanning TM domains. We speculate that this truncation triggers the nonsense-mediated decay of defective mRNA, which is the most likely reason for the loss of functional integrity of the GHRHR and hormonal signaling. Indeed, this phenomenon can be correlated with reduced or absent GH levels in patient serum and is consistent with the extremely reduced levels of serum GH in affected individuals observed in our hormone analysis.

In expression studies of truncated and chimeric epitope-linked GHRH receptors (i.e., GHRHR), DeAlmeida and Mayo (79) first identified the regions vital for the interaction of the receptor with GHRH. They investigated the fate of two truncated receptors; GHRH-delta-N, without the N-terminal domain (the region between the signal sequence and the first TM domain) and GHRH-delta-C, with trimmed downstream TM domains. In both experiments, they found diminished receptor–ligand interactions, showing that neither the N-terminus extracellular domain nor the membrane-spanning C-terminus domains were independently capable of interacting with GHRH. Instead, it was determined that although the N-terminal extracellular domain is necessary for ligand binding, the transmembrane domains and associated extracellular loop regions of the GHRHR are vital for the specific interaction with GHRH (79). The current findings also support this notion, wherein the C-terminal deletion mutation p.Glu72* also exhibits diminished interaction of GHRHR–GHRH as presented by the reduced level of growth hormones.

The p.Glu72* mutation detected in this study was previously identified by Wajnrajch et al. in an Indian family with profound GH deficiency (55), with the patient’s phenotypes found to be similar to the “little mouse” harboring Ghrhr gene mutation(s). Furthermore, the human counterparts of the mutations in the “little mouse” model (especially Asp60Gly mutation in mouse being closest to human Glu72*), were associated with comparable phenotypic features in terms of stature and hormone level. The phenotypes of patients enrolled in our study showed considerable similarities with those reported by Wajnrajch et al.: all patients exhibited phenotypes as seen in the mouse model although they did not exhibit obesity or frontal bossing. Genetic studies, including our own, have shown that carriers of *GHRHR* mutations are asymptomatic and of normal height. In a clinical investigation of a cohort of 76 adults (aged 25–75 years) carrying a heterozygous *GHRHR* mutation, Pereira et al. (80) found no association between the GHRHR mutation and short stature. We also found that all the family members (including both wild-type and heterozygous carriers) were under 5 feet in height. However, the slightly reduced sitting height of individuals from the QGP cohort remains to be investigated.

The (p.Glu72*) nonsense mutation we identified here has also been mapped in three different South Asian families from Bombay (India), Delft (an island located between Sri Lanka and India), and Sindh (Pakistan). The relative geographic connection of these families indicates the possibility of a common origin. Wajnrajch et al. performed a microsatellite-based haplotype analysis to evaluate this hypothesis among these three apparently unconnected families from the Indian subcontinent harboring the common GHRHR mutation (p.Glu72*) (81). Their evolutionary analysis revealed that these families originated from a common ancestor between 1,350 and 2,700 years ago when the three traditionally and linguistically distinct families separated from their common ancestry (47). Our identification of the same p.Glu72* mutation in a Pakistani family increases the probability of its founder effect. Furthermore, since the family in our study originated from the southernmost part of Pakistan near the Sindh Province, our study also supports the possibility of Indo-Dravidian (or Indo-Aryan) migration (81).

## Conclusions

In the current study, we identified a nonsense mutation (p.Glu72*) of GHRHR in a consanguineous Pakistani family affected by IGHD4. Biochemical analysis revealed extremely low GH levels, suggesting functional loss of the GHRHR. We confirmed the pathogenicity of this variant by *in-silico* analysis. To the best of our knowledge, this is the fourth report of a *GHRHR* mutation NM_000823:c.G214T:p.(Glu72*), and together with previous reports, our data provide further support for the founder effect of p.Glu72* in South Asian families. Our data will help to establish the genotype–phenotype correlation in IGHD type IV and, thus, will support molecular diagnosis in clinical practice. Evaluating *GHRHR* gene integrity is an important aspect of genetic evaluation in those of short stature, as early genetic diagnosis might support precision medicine treatment options. Indeed, successful treatment, i.e., hormonal therapy based on GHRHR detection, may help maintain a normal growth pattern and would subsequently enable affected patients to live a confident life in society and avoid any secondary implications of their disease, such as delayed puberty, fertility, and physique.

## Data availability statement

The datasets presented in this study can be found in online repositories. The names of the repository/repositories and accession number(s) can be found in the article/supplementary material.

## Ethics statement

This medico-genetic study was approved by the Institutional Ethical Review Board of Gomal University D.I. Khan (IRB# 04/ERB/GU), KPK, Pakistan. Informed consent was obtained from all study participants and their parents/guardians. The patients/participants provided their written informed consent to participate in this study. Written informed consent was obtained from the individual(s) for the publication of any potentially identifiable images or data included in this article.

## Author contributions

SA, MA, IAhma, and ShA assisted with the patient recruitment, conducted the experiments, and performed the data analysis. SWA performed the bioinformatic analysis. IAhme, SN, and MZ performed the sequence data analysis. MK, AAA, and KF conceptualized and supervised the study, obtained funding to support the study, and drafted the manuscript. All authors contributed to and approved the final version of this manuscript.
